# Evaluation of the Biodistribution of Serinolamide-Derivatized C_60_ Fullerene

**DOI:** 10.3390/nano10010143

**Published:** 2020-01-13

**Authors:** Nicholas G. Zaibaq, Alyssa C. Pollard, Michael J. Collins, Federica Pisaneschi, Mark D. Pagel, Lon J. Wilson

**Affiliations:** 1 Department of Chemistry and Smalley-Curl Institute, Rice University, 6100 Main St, Houston, TX 77005, USA; zaibaqn@gmail.com (N.G.Z.); acp4@rice.edu (A.C.P.); mjc178@pitt.edu (M.J.C.); 2 Department of Cancer Systems Imaging, MD Anderson Cancer Center, 1881 East Rd, Houston, TX 77054, USA; fpisaneschi@mdanderson.org

**Keywords:** fullerene, serinolamide, biodistribution, pharmacokinetics, PET

## Abstract

Carbon nanoparticles have consistently been of great interest in medicine. However, there are currently no clinical materials based on carbon nanoparticles, due to inconsistent biodistribution and excretion data. In this work, we have synthesized a novel C_60_ derivative with a metal chelating agent (1,4,7-Triazacyclononane-1,4,7-triacetic acid; NOTA) covalently bound to the C_60_ cage and radiolabeled with copper-64 (t_1/2_ = 12.7 h). Biodistribution of the material was assessed in vivo using positron emission tomography (PET). Bingel-Hirsch chemistry was employed to functionalize the fullerene cage with highly water-soluble serinolamide groups allowing this new C_60_ conjugate to clear quickly from mice almost exclusively through the kidneys. Comparing the present results to the larger context of reports of biocompatible fullerene derivatives, this work offers an important evaluation of the in vivo biodistribution, using experimental evidence to establish functionalization guidelines for future C_60_-based biomedical platforms.

## 1. Introduction

Recently, nanoparticles such as silicon nanoparticles [1], superparamagnetic iron oxide nanoparticles [2], and carbon nanotubes [3,4,5] have been studied extensively for biomedical applications as they constitute a promising platform upon which therapeutic, imaging, and/or targeting agents can be loaded [6,7,8]. Specifically, C_60_ fullerene shows promise for biomedical applications because this multi-functional platform also has a variety of inherent advantages [9]. C_60_ is chemically versatile, and many synthetic strategies of derivatization have already been developed [10,11,12,13,14,15,16], which provides great flexibility when designing a C_60_ engrafted with therapeutics. Inherently, C_60_ has also shown strong antioxidant behavior through its ability to quench free radicals [17,18] and has been observed to nearly double the lifespan of rodents, presumably because of this radical-scavenging property [19,20]. Conversely, certain C_60_ derivatives can generate reactive oxygen species under light irradiation, allowing these derivatives to be used for photodynamic therapy (PDT) against cancer [21,22]. Furthermore, C_60_ can be chemically decorated with molecular targeting agents [23,24], imaging agents [25,26,27], and anticancer drugs [24,28,29,30,31] to be used as a therapeutic delivery vector, a diagnostic agent, or a combination of the two (known as a theranostic agent).

**Figure 1:**
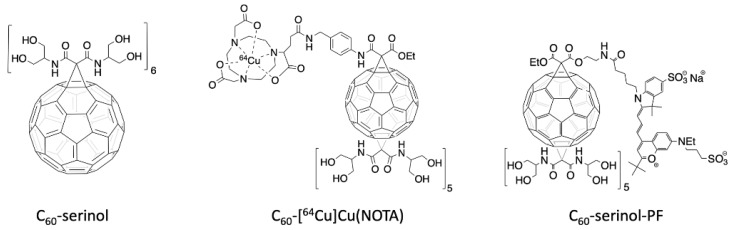
Structure of the C_60_-serinol (left). Structure of the radioactive C_60_-NOTA conjugate, radiolabeled with copper-64 (C_60_-[^64^Cu]Cu(NOTA), middle). Structure of the fluorescently-labeled C_60_-serinol conjugate with PromoFluor 633 (C_60_-serinol-PF, right).

Due to these advantageous properties of C_60_, we were inspired to design a platform that could be used as drug delivery system, to explore the potential of C_60_ as a multimodal therapeutic agent. We chose to functionalize C_60_ with the serinolamide moiety used to solubilize clinical iodobenzene X-ray contrast agents [32]. In a compound called C_60_-serinol, six malonate groups containing these serinolamide moieties surround the fullerene core in an octahedral arrangement (Figure 1, left), which affords the material with high water solubility and low cytotoxicity, making it a desirable starting material for further functionalization [33,34,35]. Like other hexakisadducts of C_60_, C_60_-serinol is monoisomeric, which helps to ensure a low polydispersity index that is necessary for clinical translation. C_60_-serinol materials have been shown to pass through the nuclear membranes of liver cancer cells [34] and has been used to deliver DNA to mouse fibroblasts and marrow stromal cells [36]. In addition, when derivatized with paclitaxel, C_60_-serinol can kill cancer cells in vivo without producing the weight loss associated with other formulations [37]. These results demonstrate C_60_-serinol’s potential to serve as a drug delivery platform, as well as the potential to meliorate the negative side effects of chemotherapy.

An important aspect of designing drug delivery platforms is the study of the biodistribution and excretion profile of the compound. Thus far, the biodistribution profiles of C_60_ reported in the literature, including C_60_-serinol, are discordant [38,39,40,41]. This work aims to reevaluate the in vivo behavior of this nanomaterial non-invasively, using the imaging technique positron emission tomography (PET). Understanding how C_60_-serinol behaves in vivo is a valuable step toward developing C_60_-derived materials for biomedical applications.

PET is routinely used in the clinic and can image whole organisms non-invasively with high sensitivity that accurately quantifies the distribution of the radioactive radiopharmaceutical in various organs. Of the many radionuclides currently used for PET imaging, we have employed copper-64 because of its relatively low cost and long 12.7 h half-life, which permits imaging at later time points post-injection [42,43]. To chelate copper-64 for radiolabeling C_60_, we have synthesized a C_60_-serinol derivative (Figure 1, center), with five malonates carrying serinolamide groups and a covalently linked NOTA chelate. [^64^Cu]Cu(NOTA) has been shown to be stable under biological conditions [44]. The biodistribution data of C_60_-[^64^Cu]Cu(NOTA), collected herein by PET, are then compared to our previously reported data of fluorescently-labeled C_60_-serinol (C_60_-serinol-PF, Figure 1, right), as well as other C_60_ derivatives reported in the literature.

## 2. Materials and Methods 

### 2.1. Synthesis and Characterization of C_60_-Cu(NOTA) 

All solvents and reagents were purchased from commercial sources and used as received. All synthetic reactions were performed under argon, unless otherwise stated. Matrix-assisted laser desorption ionization mass spectrometry (MALDI-MS) was performed using an Autoflex MALDI ToF mass spectrometer (Bruker Corporation, Billerica, MA, USA). ^1^H nuclear magnetic resonance (NMR) was performed using a 400 MHz NMR spectrometer (Bruker Corporation, Billerica, MA, USA). Fourier Transform Infrared (FTIR) spectroscopy was performed using a Nicolet 6700 FTIR spectrometer with an attenuated total reflectance (ATR) attachment (Thermo Fisher Scientific, Waltham, MA, USA). Purification of most intermediates was performed using a 971-FP Flash Purification System (Agilent Technologies, Inc., Santa Clara, CA, USA). All MS, ^1^H NMR, and IR spectra are included in the ESI. The hydrodynamic diameter and aggregate diameter of the C_60_-NOTA conjugate were determined by dynamic light scattering (DLS) and atomic force microscopy (AFM), respectively. The zeta-potential (ξ-potential) of the C_60_-NOTA and C_60_-Cu(NOTA) conjugates were determined as well. The DLS and ξ-potential measurements were conducted using a Zetasizer Nano system (Malvern Instruments, Malvern, UK). AFM images were taken using a MultiMode AFM-2 instrument (Digital Instruments, Santa Barbara, CA, USA). 

High performance liquid chromatography (HPLC) was performed using 0.1% trifluoroacetic acid (TFA) in water (solvent A) and 0.1% TFA in acetonitrile (solvent B). Purification was performed on a 1260 Infinity II preparative HPLC (Agilent Technologies, Inc., Santa Clara, CA, USA) with the following method: 5% solvent B (in solvent A) to 30% solvent B over 15 min, then 30% solvent B to 95% solvent B over the next 15 min with a 15 mL/min flow rate. A Luna^®^ C18 5 μm column with 21.2 × 250 mm dimensions (Phenomenex, Torrance, CA, USA) was used. Analytical HPLC was performed on an Agilent 1100 Series instrument using 5% solvent B to 95% solvent B over 15 min with a 1 mL/min flow rate. An XBridge^®^ C18 3.5 μm column with 4.6 × 250 mm dimensions (Waters Corp., Milford, MA, USA) was used. 

*Synthesis of Compound***1**: Benzyl *N*-[(4-aminophenyl) methyl]carbamate (2.00 g, 7.80 mmol), ethyl hydrogen malonate (1.03 g, 7.80 mmol), and N, N-diisopropylethylamine (Hünig’s base; 1.50 mL, 8.58 mmol) were dissolved in dry dichloromethane under argon at 0 °C. Diisopropylcarbodiimide (DIC; 1.25 mL, 7.96 mmol) was added dropwise over 10 min. The reaction was stirred and allowed to reach room temperature over 24 h. The solution was then dried by rotary evaporation that yielded an orange oil. Toluene was added to the oil until a white precipitate formed that was removed by vacuum filtration and discarded. The filtrate was purified by column chromatography using a gradient of dichloromethane/methanol as the eluent. The second fraction was then recrystallized from dichloromethane using hexanes, forming compound **1** as white needle-like crystals (0.376 g, 13% yield). m.p.: 108−109 °C. IR ν_max_ (neat): 3331, 3300, 2976, 1736, 1686, 1653, 1530, 1265, 1243, 1133, 1034, 971 cm^−1^. ^1^H NMR (400 MHz, CDCl_3_): δ 9.23 (s, 1H), 7.48−7.44 (m, 2H), 7.35−7.27 (m, 5H), 7.22−7.17 (m, 2H), 5.34 (t, *J* = 5.9 Hz, 1H), 5.11 (s, 2H), 4.30 (d, *J* = 5.9 Hz, 2H), 4.22 (q, *J* = 7.2 Hz, 2H), 3.42 (*s*, 2H), 1.30 (t, *J* = 7.2 Hz, 3H). MALDI-MS: *m*/*z* calculated for C_20_H_22_N_2_O_5_, 370.398; found, 371.128 [M^+^], 393.145 [M + Na^+^], 409.203 [M + K^+^].

*Synthesis of Compound***2**: C_60_ (1.00 g, 1.39 mmol) was dissolved in 800 mL of dry toluene by bath sonication. Compound **1** (0.645 g, 1.74 mmol) and carbon tetrabromide (0.922 g, 2.78 mmol) were also added to the reaction mixture. A 1 M solution of 1,8-diazabicycloundec-7-ene (DBU) in toluene (1.94 mL, 1.94 mmol) was then added in two aliquots 10 min apart, and the reaction was stirred for 4.5 h at room temperature. A black precipitate formed during the reaction that was removed by vacuum filtration and discarded before the reaction mixture was purified by column chromatography using a gradient of toluene/ethyl acetate as the eluent. The second fraction was collected, and the solvent was removed by rotary evaporation to afford compound **2** as a dark red solid (0.121 mg, 8% yield). MALDI-MS: *m*/*z* calculated for C_80_H_20_N_2_O_5_, 1089.024; found, 1089.633 [M^+^], 1112.690 [M + Na^+^], 1127.620 [M + K^+^].

*Synthesis of Compound***3**: Compound **2** (0.048 g, 44.5 μmol) was dissolved in a 1:1 mixture of dry toluene and dry dichloromethane. *N*,*N*’-bis[2-(acetyloxy)-1-[acetyloxy)methyl]ethyl]-malonamide (protected serinolamide malonate; 0.187 g, 0.448 mmol), which was previously synthesized according to the method reported by Wharton et al. [33], was dissolved in dry dichloromethane and added to the reaction flask. Carbon tetrabromide (0.221 g, 0.667 mmol) was then added to the reaction mixture followed by DBU (0.245 mL, 0.245 mmol) in 6 mL of toluene dropwise over 6 h. The reaction mixture was purified by column chromatography using a gradient of chloroform/methanol as the eluent. The first fraction was collected, and the solvent was removed by rotary evaporation giving compound **3** as a light red solid (0.128 g, 91% yield). IR ν_max_ (neat): 3294, 3064, 2958, 1736, 1663, 1538, 1366, 1219, 1041 cm^−1^. MALDI-MS: *m*/*z* calculated for C_165_H_140_N_12_O_55_, 3170.919; found, 3169.637 [M^+^], 3192.630 [M + Na^+^]. 

*Synthesis of Compound***4**: Trifluoroacetic acid (TFA; 3.10 μL, 40.5 μmol) was dissolved in isopropyl alcohol to give a 1.04 M solution. Compound **3** (0.128 g, 40.5 μmol) was then added along with methanol until everything was dissolved. Pd/C (0.27 g) was added to the reaction mixture, and the vial was placed in a pressurized vessel with 10.2 atm of H_2_. The reaction mixture was stirred at room temperature for about 12 h. The mixture was then filtered by vacuum filtration to remove the Pd/C, and the solvent was removed by rotary evaporation to give compound **4** as a red solid without further purification (0.091 g, 74% yield). MALDI-MS: *m*/*z* calculated for C_157_H_134_N_12_O_53_, 3036.787; found, 3037.912 [M^+^], 3058.515 [M + Na^+^].

*Synthesis of Compound***5**: Compound **4** (0.091 g, 29.9 μmol), 4-(4,7-bis(2-(*tert*-butoxy)-2-oxoethyl)-1,4,7-triazonan-1-yl)-5-(tert-butoxy)-5-oxopentanoic acid (NODAGA-t-Bu; 0.016 g, 29.9 μmol), and Hünig’s base (5.73 μL, 32.9 μmol) were dissolved in dry dichloromethane at 0 °C. DIC (4.78 μL, 30.5 μmol) was then added dropwise over 10 min. The reaction was stirred for 12 h and warmed to room temperature as the reaction proceeded. The solvent was removed by rotary evaporation to afford compound **5** as a red solid without further purification. (0.093 g, 87% yield). IR ν_max_ (neat): 3323, 2979, 1739, 1680, 1538, 1368, 1224, 1150, 1046 cm^−1^. MALDI-MS: *m*/*z* calculated for C_184_H_181_N_15_O_60_, 3562.463; found, 3564.097 [M^+^].

*Synthesis of Compound***6***(C_60_-NOTA conjugate)*: Compound **5** (0.091 g, 25.5 μmol) was dissolved in 10 mL of p-dioxane. 12 M HCl (0.833 mL) was added to the solution, and it was stirred for 5 d at room temperature under atmospheric conditions. The reaction mixture separated into two phases for the first 24 h. Distilled water was added at 48 h until the reaction mixture became homogeneous. After the fifth day, the mixture was dried using rotary evaporation, and the product was dissolved in distilled water and purified using dialysis centrifuge tubes with a 3500 molecular weight cut-off membrane in water that were stirred at 3600 rpm for 60 min. The dialysate was collected and subjected to additional dialysis using a 2000 molecular weight cut-off membrane cartridge in distilled water for two weeks. After drying by lyophilization, compound **6** was afforded as an orange solid (7.16 mg, 11% yield). The identity and purity of the compound was confirmed by analytical HPLC (retention time = 7.6 min) and ^1^H NMR through confirmation that the acetate protecting groups on the serinolamide moieties had been completely hydrolyzed (absence of peak at 2.08 ppm). IR ν_max_ (neat): 3268, 3063, 2943, 2880, 1652, 1531, 1460, 1283, 1040 cm^−1^. The C_60_-NOTA conjugate was then dissolved in distilled water (<1 mg/mL) and analyzed using dynamic light scattering (DLS) to determine the hydrodynamic diameter. The C_60_-NOTA solution was added to a disposable cuvette with a 1 cm path length and allowed to equilibrate for two minutes prior to sizing measurements. The obtained intensity profiles were used, and a correlation function was used to determine the intensity-weighted hydrodynamic diameter of samples. All measurements for a particular sample were combined to provide a statistical average at all sizes measured and plotted as an intensity profile. The ξ-potential of the C_60_-NOTA conjugate was measured using disposable capillary cells using the same solution used to measure the hydrodynamic diameter. AFM images were obtained by making a dilute solution of the C_60_-NOTA conjugate in isopropanol and drop-casting 2–4 drops of solution onto a mica substrate. The samples were analyzed in tapping mode with 512 lines at 0.5 Hz.

*Synthesis of C_60_-Cu(NOTA)*: CuCl_2_ (1.30 mg, 10.0 μmol) was dissolved in 1 mL of 0.1 M NaOAc buffer, pH = 6. C_60_-NOTA (6.40 mg, 2.50 μmol) was added in one portion under atmospheric conditions. The reaction mixture was heated to 45 °C, stirred overnight, and then purified using preparative HPLC to afford the title compound as an orange solid. The ξ-potential of the C_60_-Cu(NOTA) conjugate was measured as described above. 

### 2.2. Radiochemistry Studies

All solvents and reagents were purchased from commercial sources and used as received. Water was deionized using a Milli-Q integral water purification system (MilliporeSigma, Burlington, MA, USA). [^64^Cu]CuCl_2_ was produced from a 16 MeV proton/deuteron GE PETtrace 10 cyclotron (GE Healthcare, Chicago, IL, USA) using an EDS/PTS solid target station (Comecer S.p.A., Castel Bolognese, Italy) in the Cyclotron Radiochemistry Facility at the MD Anderson Cancer Center. Radioactivity was detected during HPLC using a Bioscan Model 106 detector interfaced with the analytical HPLC through an Agilent Interface 35900E (same analytical and preparative instruments, methods, and columns as described in previous sections).

*Radiolabeling of C_60_-[^64^Cu]Cu(NOTA)*: Cyclotron-produced [^64^Cu]CuCl_2_ in 0.1 M HCl (2.69 mCi, 3 μL) was added to 100 μL of 0.1 M NaOAc buffer, pH = 5.6. C_60_-NOTA (80 μg, 31 nmol) in 40 μL of water was added, and the reaction was heated to 37 °C for 60 min. Reaction completion was confirmed with analytical HPLC. The reaction mixture was loaded onto a PD-10 column (GE Healthcare, Chicago, IL, USA) and eluted with 5 mL of 1 × phosphate buffered saline (PBS) in 500 μL fractions. A decay-corrected radiochemical yield of 49 ± 5% (n = 4) was obtained. C_60_-[^64^Cu]Cu(NOTA) was obtained in 98% purity with a molar activity of 1.35–3.06 GBq/μmol.

*Radiolabeling of [^64^Cu]Cu(NOTA)*: Cyclotron-produced [^64^Cu]CuCl_2_ in 0.1 M HCl (1.027 mCi, 1 μL) was added to 100 μL of 0.1M NaOAc buffer, pH = 5.6. NOTA (40 μg, 130 nmol) in 40 μL of water was added, and the reaction was heated to 37 °C for 45 min. Reaction completion was confirmed with analytical HPLC. The reaction mixture was buffered with 300 μL of 1X PBS and injected into mice without further purification. [^64^Cu]Cu(NOTA) was obtained in >99% purity with a molar activity of 0.268–0.601 GBq/μmol.

*Shelf and Serum Stability Studies*: C_60_-[^64^Cu]Cu(NOTA) (120 μCi, 200 μL) was incubated with 200 μL of human serum at 37 °C. After 20 and 48 h, the sample was analyzed with analytical HPLC to determine integrity. A shelf stability test with C_60_-[^64^Cu]Cu(NOTA) (529 μCi, 500 μL) and [^64^Cu]Cu(NOTA) (235 μCi, 90 μL) in PBS was also conducted at room temperature and at 4 °C. After 24 and 48 h, the samples were analyzed with analytical HPLC to determine integrity.

### 2.3. PET Imaging and Metabolism Studies

PET and CT scans were performed using a small animal Albira PET/SPECT/CT scanner (Bruker Corporation, Billerica, MA, USA). PET images were analyzed using PMOD Version 3.505 (PMOD Technologies Ltd., Zürich, Switzerland). PET/CT maximum intensity projections were constructed using Inveon Research Workplace (Siemens Medical Solutions USA, Inc., Malvern, PA, USA). Statistical analysis was performed on GraphPad Prism 8 (GraphPad Software, San Diego, CA, USA).

*PET Image Acquisition*: All mice were manipulated in accordance to the MD Anderson Cancer Center’s IACUC guidelines. Mice were anesthetized with 2% isoflurane using oxygen as a carrier. For both C_60_-[^64^Cu]Cu(NOTA) and [^64^Cu]Cu(NOTA), 6 male nude mice were injected on the bed with ~200 μCi of activity in 150 μL of saline through a tail vein catheter. At the start of injection, a 20-min dynamic PET scan was acquired using the Albira PET/SPECT/CT scanner with a 15 cm field of view (FOV). The actual injected dose was calculated by measuring the pre- and post-injection activity in the syringe using a CRC-15R dose calibrator (Capintec, Inc., Florham Park, NJ, USA). The data were binned into 20-s time frames for the first 5 min, 60-s frames for the next 5 min, and 5-min frames for the next 10 min. Static PET images were then recorded at 3 h and 24 h post-injection (p.i.) for 10 min. A static PET image at 48 h p.i. was recorded for 20 min for C_60_-[^64^Cu]Cu(NOTA) only. Following all PET scans, two 3 min CT scans were performed to cover the whole mouse body with a total FOV of 12.6 cm (400 μA, 45 kV, 250 projections).

The PET images were reconstructed using the Maximum Likelihood Expectation Maximization method with 12 iterations. The CT images were reconstructed using the Filtered Back Projection method. Scatter, randoms, decay, and attenuation corrections were applied through the Albira software. Volumes-of-interest (VOIs) were drawn for the major organs on the decay-corrected PET images using PMOD, and injected doses were used to calculate percent of injected dose per cubic centimeter of tissue (%ID/cc). Errors in the averaged %ID/cc were reported as standard error of the mean (SEM). A non-linear regression analysis of the dynamic PET data for both C_60_-[^64^Cu]Cu(NOTA) and [^64^Cu]Cu(NOTA) was performed and fitted to a two-phase decay model to determine the blood half-life of each compound.

*Metabolism Stability Studies*: 179 μCi, 204 μCi, and 205 μCi of C_60_-[^64^Cu]Cu(NOTA) were injected through a tail vein catheter into three nude mice under anesthesia with 2% isoflurane using oxygen as a carrier. The mice remained anesthetized, and blood and urine were collected from each mouse after 20 min and rapidly cooled on dry ice to halt metabolism. The urine was injected directly onto the analytical HPLC. The blood was centrifuged with acetonitrile, and the serum/acetonitrile layer was injected onto the analytical HPLC. Additional urine was collected from anesthetized mice at 24 h and injected directly onto the analytical HPLC.

## 3. Results

### 3.1. Synthesis and Characterization of C_60_-NOTA Conjugate

**Figure 2:**
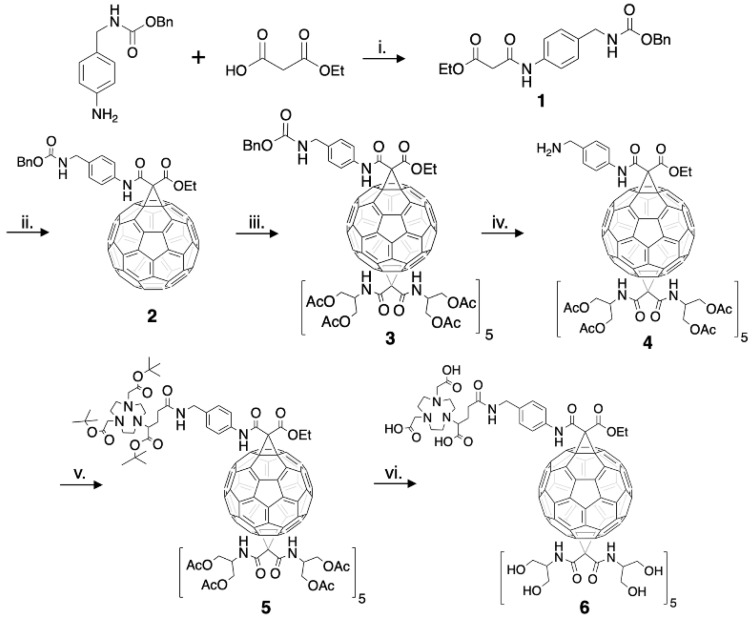
Complete synthesis of the C_60_-NOTA conjugate (compound **6**). **i**: DIC, Hünig’s base, 0 °C → RT, 12 h, 13% yield. **ii**: C_60_, CBr_4_, DBU, RT, 4.5 h, 8% yield. **iii**: *N*,*N*’-bis[2-(acetyloxy)-1-[acetyloxy)methyl]ethyl]-malonamide, CBr_4_, DBU, RT, 6 h, 91% yield. **iv**: H_2_, Pd/C, RT, 12 h, 74% yield. **v**: NODAGA-t-Bu, DIC, Hünig’s base, 0 °C → RT, 12 h, 87% yield. **vi**: 1 N HCl, dioxane, RT, 4-5 d, 11% yield.

The structure of a C_60_-serinol derivative amenable to PET imaging was constructed to include a chelate that could be radiolabeled with copper-64. The bifunctional chelate derivative of NOTA (2-(4,7-bis(carboxymethyl)-1,4,7-triazonan-1-ylpentanedioic acid, or NODAGA) was chosen as NOTA has been shown to exhibit high kinetic stability for copper in radiochemical studies when compared to other chelates [44,45,46]. The C_60_-NOTA conjugate was synthesized according to the procedure outlined in Experimental Section 2.1 (Figure 2). Compound **1** was formed using a DIC-mediated amide bond formation reaction between benzyl *N*-[(4-aminophenyl)methyl]carbamate and ethyl hydrogen malonate in the presence of Hünig’s base. Formation of compound **2** utilized a Bingel-Hirsch reaction with the derivatized malonate to replace one 6–6 double bond of fullerene with a cyclopropane ring after in situ formation of the bromomalonate. The monoadduct was easily separated from unreacted C_60_ and the bisadduct using column chromatography. A subsequent Bingel-Hirsch reaction was then performed with a large excess of the protected serinolamide compared to compound **2** to control the number of groups added to the surface. Slow DBU addition and long reaction times also led to the formation of higher adduct products. The hexakisadduct (compound **3**) showed a favorable formation using these conditions; however, a small amount of pentakisadduct occasionally remained in the final product. The benzyloxy carbamate protecting group was then removed from the malonate linker via hydrogenolysis, and the tert-butyl protected NOTA chelate was added through another DIC-mediated coupling in the presence of Hünig’s base to give compound **5**. Finally, a global acid deprotection removed the tert-butyl and acetate protecting groups to give the final C_60_-NOTA conjugate (compound **6**).

**Figure 3:**
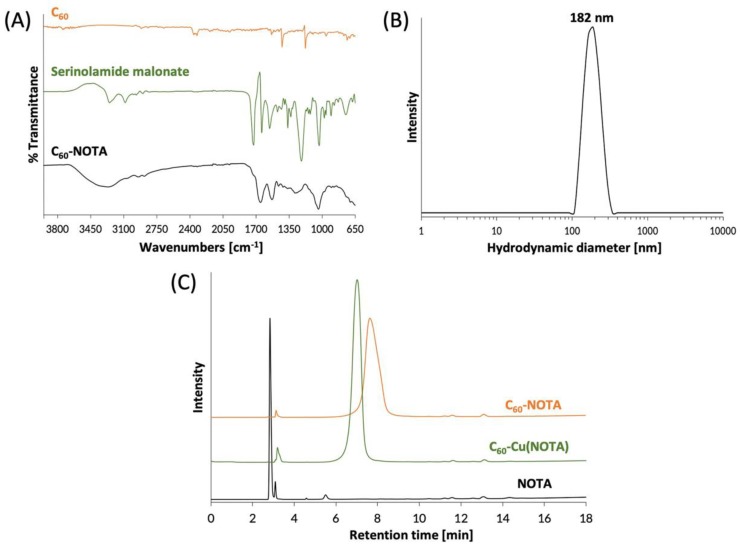
Chemical characterization of C_60_-NOTA. (**A**) FTIR spectra of naked C_60_ (orange), the deprotected serinolamide malonate compound that is conjugated to the C_60_ cage (green), and the C_60_-NOTA conjugate (black); (**B**) DLS spectrum showing the hydrodynamic diameter of C_60_-NOTA; (**C**) HPLC chromatograms of C_60_-NOTA (orange), C_60_-Cu(NOTA) (green), and NOTA alone (black). Peak at 3.1 min is an impurity in the commercial NOTA compound that did not affect radiochemistry.

The C_60_-NOTA material (compound **6**) was characterized by FTIR spectroscopy to confirm introduction of functional groups to the C_60_ cage. The FTIR spectrum of C_60_-NOTA was distinct from that of the naked C_60_ and the serinolamide groups (Figure 3A). C_60_-NOTA contained peaks at 1410, 1652, 2943, and 3268 cm^−1^ representing the carboxylic acid O-H stretch from NOTA, the C=C stretch of the C_60_ cage, the N-H stretch from serinolamide and NOTA linker malonates, and the O-H stretch from the many hydroxyls on the serinolamide groups, respectively. Dynamic light scattering (DLS) was employed to measure the hydrodynamic diameter of the nanostructure in solution (Figure 3B). The DLS measurements showed that C_60_-NOTA conjugate forms uniform aggregates in water with an average diameter of 182 nm and an average polydispersity index of 0.27. These results agreed with the aggregate size that C_60_-serinol itself has shown to form in aqueous solution (median aggregate size 100–200 nm) [47]. These DLS results were also in good agreement with the aggregate size found in the solid state as determined by atomic force microscopy (AFM) images (Figure S11) which showed the average aggregate size to be 195 nm.

The C_60_-NOTA conjugate was then chelated with nonradioactive Cu^2+^ to demonstrate the chelating ability of NOTA while appended to the C_60_ cage. Following a literature procedure [48], the material was labeled with CuCl_2_ dissolved in an sodium acetate buffer at pH 6, which was then stirred overnight at 45 °C. The metallated conjugate, C_60_-Cu(NOTA), the unmetallated conjugate, C_60_-NOTA, and the unmetallated chelate, NOTA, were characterized by HPLC and were found to have different retention times (2.8 min for NOTA, 7.0 min for C_60_-Cu(NOTA) and 7.6 min for C_60_-NOTA; Figure 3C). These results demonstrated that conjugation of NOTA to C_60_ was successful and that the chelate successfully bound copper. Furthermore, the HPLC plots show there is no free NOTA within the C_60_-NOTA material, which would have been problematic during the radiolabeling because NOTA and C_60_-NOTA would compete for chelation with copper-64. The ζ-potential of C_60_-NOTA and C_60_-Cu(NOTA) were also investigated to determine the surface charge on the conjugate, which can affect the biodistribution of the material [49]. The ζ-potential of C_60_-NOTA and C_60_-Cu(NOTA) were −22.5 mV and −11.4 mV, respectively, which agrees well with ζ-potential measurements of C_60_-serinol and other water soluble C_60_ materials [39,47,50].

### 3.2. Radiolabeling of C_60_-NOTA

**Figure 4:**
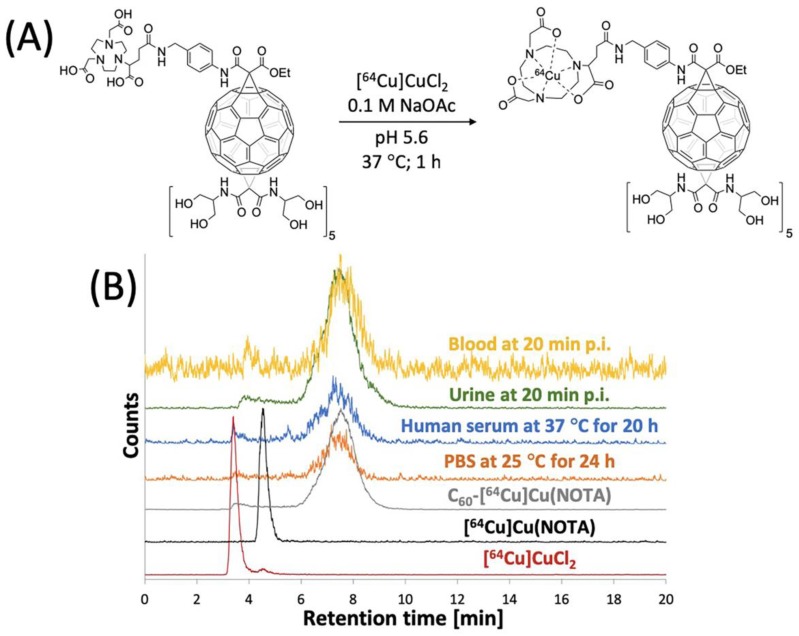
Radiolabeling of C_60_-NOTA. (**A**) Radiolabeling reaction scheme; (**B**) Radio-HPLC chromatograms of [^64^Cu]CuCl_2_ (red), [^64^Cu]Cu(NOTA) (black), and C_60_-[^64^Cu]Cu(NOTA) (gray), C_60_-[^64^Cu]Cu(NOTA) challenged against PBS at 25 °C for 24 h (orange), C_60_-[^64^Cu]Cu(NOTA) challenged against human serum at 37 °C for 20 h (blue), mouse urine sample at 20 min p.i. (green), and mouse blood sample at 20 min p.i. (yellow).

The C_60_-NOTA conjugate was radiolabeled with copper-64, and its radiochemical purity and stability in biological media were evaluated. The conjugate was radiolabeled with copper-64 in a sodium acetate buffer (pH 5.6) at 37 °C for 1 h and tracked by analytical radio-HPLC (Figure 4). C_60_-[^64^Cu]Cu(NOTA) exhibited a different retention time than [^64^Cu]Cu(NOTA) and free copper-64 on radio-HPLC. After purification, a decay-corrected yield of 49 ± 5% (n = 4) was obtained, and the radio-HPLC chromatograms show that C_60_-[^64^Cu]Cu(NOTA) had a radiochemical purity of 97%. Furthermore, the metal ion stability of C_60_-[^64^Cu]Cu(NOTA) was challenged in PBS for 24 and 48 h at 25 °C and human serum for 20 and 48 h at 37 °C (Figures S13 and S14). C_60_-[^64^Cu]Cu(NOTA) proved to be 93% and 95% stable in PBS at 24 h and 48 h, respectively. In addition, C_60_-[^64^Cu]Cu(NOTA) was 91% and 89% stable in human serum at 20 h and 48 h, respectively.

### 3.3. PET Imaging of C_60_-[^64^Cu]Cu(NOTA)

**Figure 5:**
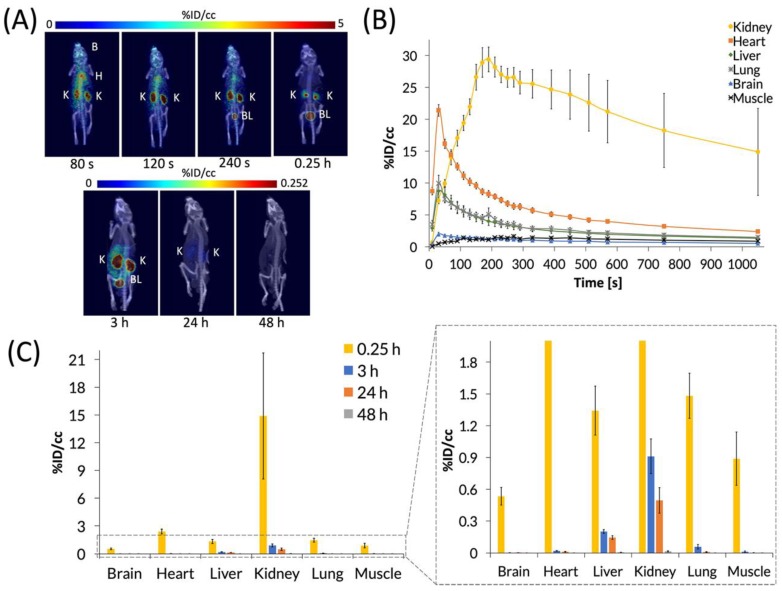
PET images and biodistribution of C_60_-[^64^Cu]Cu(NOTA). (**A**) Whole-body dynamic and static PET images acquired at various time points post-injection (p.i.). Organs labeled are brain (B), heart (H), kidney (K), and bladder (BL); (**B**) Time activity curve (TAC) of 20 min dynamic scan showing initial biodistribution and rapid decrease in uptake in most organs; (**C**) Quantification of radioactivity in VOIs showing accumulation in various organs with a close-up view of the graph to highlight lower accumulation. Data represent mean ± SEM, n = 6.

C_60_-[^64^Cu]Cu(NOTA) was then administered to mice by tail-vein injection of ~200 μCi in 150 μL of saline, and mice were imaged by PET/CT. First, a 20-min dynamic scan was acquired for each animal, followed by static scans that were taken at 3, 24, and 48 h p.i. (Figure 5). Representative PET images (Figure 5A) and the time activity curve (TAC) of the dynamic scan (Figure 5B) show that C_60_-[^64^Cu]Cu(NOTA) accumulated in tissues as it circulated and then decreased rapidly in all organs except the kidneys. The conjugate material cleared rapidly from the heart, liver, and lungs over the first 6 min, while showing negligible uptake in the brain and muscle. At 20 min p.i., the kidneys and bladder still had the highest uptake of C_60_-[^64^Cu]Cu(NOTA), as seen in the PET image for that time point. The amount of radioactivity significantly decreased at 3 and 24 h p.i. for all organs, and there was negligible uptake for any organ at 48 h, indicating the material had effectively cleared from the mice (Figure 5C). These biodistribution data resemble trends seen for X-ray contrast agents functionalized with serinolamide groups including the rapid renal clearance [32].

Because PET detects signal from the radioactive copper-64 ion and not from the fullerene compound itself, it was important to assess C_60_-[^64^Cu]Cu(NOTA) stability in vivo. To answer this question, blood and urine samples from mice were collected at 20 min p.i. and analyzed using radio-HPLC to determine the integrity of the excreted material (Figure 4B). These data indicated that the conjugate is highly stable in vivo with 97% and 92% retention of copper in urine and blood, respectively. Furthermore, there was no evidence that any [^64^Cu]Cu(NOTA) was cleaved from the fullerene conjugate, which suggested the material was excreted without significant modification.

### 3.4. PET Imaging of [^64^Cu]Cu(NOTA)

**Figure 6:**
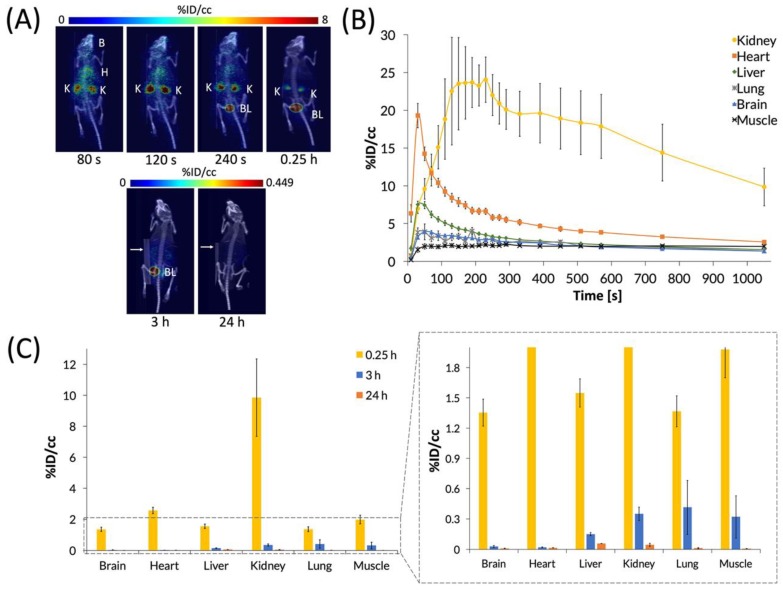
PET images and biodistribution of [^64^Cu]Cu(NOTA). (**A**) Whole-body dynamic and static PET images acquired at various time points post-injection (p.i.). Organs labeled are brain (B), heart (H), kidney (K), and bladder (BL). White arrows for 3 and 24 h p.i. images highlight a breathing pad that was placed under the mice during imaging. The radioactivity was negligible at 48 h p.i., so it could not be imaged and quantified accurately, suggesting that [^64^Cu]Cu(NOTA) was no longer present in vivo at 48 h p.i.; (**B**) The time activity curve (TAC) of a 20 min dynamic scan showing initial biodistribution and rapid drop-off in uptake in most organs; (**C**) Quantification of radioactivity in various organs with a vertically expanded view of the graph to highlight lower accumulation. Data represent mean ± SEM, n = 6.

After characterizing the biodistribution of the C_60_-[^64^Cu]Cu(NOTA) conjugate, we sought to examine how the biodistribution of [^64^Cu]Cu(NOTA) compares to that of the C_60_ conjugate (Figure 6). These studies showed that [^64^Cu]Cu(NOTA) has a similar biodistribution profile to C_60_-[^64^Cu]Cu(NOTA) with significant uptake in the kidneys at 20 min p.i. compared to other major organs at the same time point. However, accumulation decreased rapidly at 3 and 24 h p.i. Although mice were imaged at 48 h p.i., the detected signal was negligible. This evidence indicated that most of the material was cleared by renal excretion between 20 min and 3 h p.i. These data are supported by previous biodistribution studies of other small-molecule, macrocyclic copper chelates of similar structure, such as DOTA and TETA, which were also found to be almost completely cleared within 24 h [51].

We also performed a non-linear regression analysis of the heart TAC data of both compounds using a two-phase decay model, which produced a *p* value of < 0.0001 by an extra sum-of-squares F test. The two-phase decay model consists of a distribution phase and an elimination phase. The fast distribution phase describes the rapid circulation of the material from the plasma to highly-perfused tissues, and the slow elimination phase describes the clearance of the material from the plasma and tissues through excretion. From this analysis, a blood half-life (t_1/2_) and rate constants (α, β) for each phase were determined along with the clearance rate (CL) and the area under the curve (AUC), which represents a normalized volume of distribution (Table 1).

## 4. Discussion

C_60_-[^64^Cu]Cu(NOTA) was produced in high yields and radiochemical purity, was found to retain copper-64 higher than 90% in in vitro and in vivo studies, and was administered intravenously in mice to assess its biodistribution by PET/CT. A dynamic 20 min PET scan was acquired, followed by static 3, 24 and 48 h post injection follow-up scans. The radiolabeled fullerene cleared very quickly through the kidneys in the first hour post injection, with very minimal accumulation in the other organs at later time points. This was somewhat surprising as nanoparticles tend to have a hepatobiliary clearance pathway. Our group and others have shown that carbon nanotubes and other types of nanoparticles typically localize to the lungs, liver, and spleen [1,3,5,52,53,54]. Mechanistically, as previously described by Aggarwal, et al. protein aggregation around nanoparticles increases with the more hydrophobic surface exposed, leading to macrophage uptake and excretion through reticuloendothelial system (RES) organs [55]. In our work, it is likely that coating the hydrophobic surface of C_60_ with hydrophilic serinolamide groups prevented such aggregation and allowed for C_60_-[^64^Cu]Cu(NOTA) to be excreted through the kidneys as single particles. In addition, C_60_-[^64^Cu]Cu(NOTA) did not significantly collect in the lungs or liver, which also mitigates long-term toxicity concerns of fullerenes [56].

As is the case with any nanomaterial, surface chemistry is known to play an important role in the material’s in vivo behavior [57]. For example, there have been many reports of C_60_ derivatives with less hydrophilic coverage than that of the present study, such as hydroxylated and carboxylated fullerenes, which showed greater retention in the lungs, muscle, and RES organs, in addition to longer residence times in vivo (~30 h) [26,38,41,58,59,60,61]. However, leaving some lipophilic character on the C_60_ surface can result in the penetration of certain restrictive membranes, such as the blood-brain-barrier, as was recently reported by Dugan and coworkers when administering ^14^C-labeled e,e,e-methanofullerene(60)-63-tris malonic acid (C_3_) [40]. C_3_ showed significant liver and kidney uptake at 12 and 24 h p.i., which resulted in fecal excretion as the route of clearance. These examples contrast with the C_60_-[^64^Cu]Cu(NOTA) conjugate of this work, which only showed rapid renal clearance under 3 h.

While the above examples showed studies that observed long in vivo residence times for fullerene derivatives, one study reported a fast-clearing amino-PEGylated C_60_ derivative [39]. The authors analyzed this material that had a similar diameter and surface charge as our construct, which was also radiolabeled with [^64^Cu]Cu(NOTA). While the biodistribution data reported by these authors at later time points generally agree with the results presented here, conducting a dynamic PET scan after administration of C_60_-[^64^Cu]Cu(NOTA) allowed us to study the biodistribution profile more thoroughly, which was especially important when evaluating a material that clears this quickly. Overall, these similar results to our work show that masking the hydrophobic surface of C_60_ with different hydrophilic groups can effectively lead to fast renal clearance. Furthermore, this group examined the toxicity of their material and found that the compound was not cytotoxic at 100 μg/mL exposure [39]. We have previously shown that C_60_-serinol is likewise non-cytotoxic at the same concentration [34].

From the blood half-life data (Table 1), it is evident that clearance characteristics are similar for C_60_-[^64^Cu]Cu(NOTA) and the [^64^Cu]Cu(NOTA) control agent. While the appended imaging agent has potential to influence the pharmacokinetics of C_60_-[^64^Cu]Cu(NOTA), both C_60_-[^64^Cu]Cu(NOTA) and [^64^Cu]Cu(NOTA) had similar “small molecule-like” behavior in vivo. C_60_-[^64^Cu]Cu(NOTA) clears more quickly from the blood, with distribution and elimination half-life values of 0.6436 and 7.078 min respectively, compared to reported C_60_-drug conjugates with similar hydrodynamic diameters and ξ-potentials with elimination half-life values between 200–500 min [62,63]. These C_60_-drug conjugates bound to monomethyl fumarate and tamoxifen were both made water soluble with four units of PEG (tetraethylene glycol). Another study reported an even longer t_1/2_ (1.8 x 10^4^ min), which is likely due to the hydrophobic nature of the conjugate, as it was derivatized with the hydrophobic drug docetaxel [64].

Comparing present results for C_60_-[^64^Cu]Cu(NOTA) to our previous work with a fluorescently-labeled C_60_-serinol-PF conjugate (Figure 1) [34], we found dramatically different profiles even though both nanomaterials are derivatives of C_60_-serinol. C_60_-serinol-PF was observed as predominately retaining in the tumor, kidneys, liver, and brain in a liver cancer mouse model (Hep3B) for longer than 100 h p.i. Although no tumor model was used in the present study, the C_60_-[^64^Cu]Cu(NOTA) conjugate showed little uptake in the liver at 24 h p.i. and essentially no uptake in the brain. These strikingly different biodistribution profiles raise the question as to why these two C_60_-serinol-based materials behave differently in vivo. Controls for the effects of derivatization of various nanomaterials are often lacking in the literature [61], and unfortunately, a control experiment examining the biodistribution of the PF fluorophore alone is not available. While the biodistribution pattern of C_60_-[^64^Cu]Cu(NOTA) nanomaterial is similar to that of the [^64^Cu]Cu(NOTA) imaging tag, it is quite distinguished from that of the fluorescently-labeled C_60_-serinol-PF. The biodistribution data for C_60_-serinol-PF also showed significant uptake in the heart at greater than 100 h p.i., indicating that the material was still circulating in the blood as a blood-pool agent. In fact, a recent study of C_60_-serinol-PF biotransport kinetics reported that the material does not leak from normal vasculature as it does in tumor vasculature [47], leading to the conclusion that the PF fluorophore converts the C_60_-serinol platform into a blood pool agent.

While the in vivo data presented here for C_60_-[^64^Cu]Cu(NOTA) provides evidence that C_60_-serinol could serve as an ideal platform for designing a C_60_-based biomedical material, further work should be done to tailor the biodistribution to the desired application. For example, there are 30 theoretical sites available on C_60_ for Bingel-Hirsch chemistry [65], and based on our experience, only two of those sites are required for serinolamide moieties to achieve water solubility. Therefore, reducing the hydrophilic character of C_60_-serinol, as well as further functionalizing the surface with targeting agents to deliver therapeutic cargo is likely to increase residence time in vivo and make C_60_-serinol better suited for drug delivery. It is also important to note that the biodistribution of any therapeutic cargo that C_60_-serinol would carry can have a significant impact on the delivery of that cargo. Moreover, using a C_60_-based drug delivery vehicle allows for the potential of multimodal therapy by also utilizing the attractive therapeutic properties of the C_60_ core, such as potent antioxidant activity and PDT capabilities [66], which is not possible with simply targeting small-molecule drugs or sequestering drugs within liposomes. While more work is necessary to tailor the delivery of drug cargo using C_60_-serinol, it is clear through the in vivo data for the nanostructure that it is a benign platform with ideal in vivo behavior for biomedical applications including utilizing the inherent properties of C_60_ in concert with small-molecule drugs.

## 5. Conclusions

Herein, we have presented the synthesis, characterization, radiolabeling, and PET-determined biodistribution of a highly water-soluble C_60_ derivative, based on a C_60_-serinol platform. It was demonstrated by comparing the biodistribution and pharmacokinetic parameters of C_60_-[^64^Cu]Cu(NOTA) and [^64^Cu]Cu(NOTA) that C_60_-[^64^Cu]Cu(NOTA) exhibits “small molecule-like” behavior in vivo with quick renal clearance. This study provides rationale for conducting a control imaging study for the imaging agent when performing biodistribution studies on C_60_ derivatives. In addition, using radionuclides as an imaging tag for these biodistribution studies allows for tracking in vivo with high sensitivity and accuracy using non-invasive PET imaging over time. Therefore, the work presented here provides valuable insights about the rational design of future biomedical nanoparticles and demonstrates effective methods to evaluate the biodistribution and pharmacokinetics of these interesting materials.

## Figures and Tables

**Table 1:** Pharmacokinetic Parameters for C_60_-[^64^Cu]Cu(NOTA) and [^64^Cu]Cu(NOTA) ^1^.^.^

Material	Distribution Half-Life [min]	Distribution Rate Constant (α) [min^−1^]	Elimination Half-Life [min]	Elimination Rate Constant (β) [min^−1^]	Clearance (CL) [cc/min]	Area under Curve (AUC) [%ID·min/cc]
C_60_-[^64^Cu]Cu(NOTA)	0.6436	1.077	7.078	9.793 × 10^−2^	3.161 × 10^−5^	3.164 × 10^2^
[^64^Cu]Cu(NOTA)	0.4975	1.393	8.153	8.501 × 10^−2^	3.152 × 10^−5^	3.173 × 10^2^

^1^ Two-phase decay model: R^2^ = 0.966 for C_60_-[^64^Cu]Cu(NOTA) and for [^64^Cu]Cu(NOTA).

## Supplementary Materials

The following are available online at https://www.mdpi.com/2079-4991/10/1/143/s1, Figure S1: IR of compound **1**, Figure S2: ^1^H NMR of compound **1**, Figure S3: MALDI-MS of compound **1**, Figure S4: MALDI-MS of compound **2**, Figure S5: IR of compound **3**, Figure S6: MALDI-MS of compound **3**, Figure S7: MALDI-MS of compound **4**, Figure S8: IR of compound **5**, Figure S9: MALDI-MS of compound **5**, Figure S10: ^1^H NMR of compound **6**, Figure S11: AFM of C_60_-NOTA, Figure S12: Characterization of C_60_-[^64^Cu]Cu(NOTA), Figure S13: Shelf stability study, Figure S14: Shelf stability study.
