# Supplementary material for: Evaluation of the Biodistribution of Serinolamide-Derivatized C60 Fullerene
Source: Nanomaterials (Basel). 2020 Jan 13;10(1):143. doi: 10.3390/nano10010143 (PMC7023239; doi:10.3390/nano10010143)
Supplement: Supplementary file 1 [file nanomaterials-10-00143-s001.pdf]

SUPPLEMENTARY MATERIALS

Evaluation of the Biodistribution of Serinolamide-Derivatized C_{60} Fullerene

Nicholas G. Zaibaq[1]†, Alyssa C. Pollard[1,2]†, Michael J. Collins[1,3], Federica Pisaneschi[2], Mark D. Pagel[2]*, and Lon J. Wilson[1]*

[1]Department of Chemistry and Smalley-Curl Institute, Rice University, 6100 Main St, Houston, TX 77005

[2]Department of Cancer Systems Imaging, MD Anderson Cancer Center, 1881 East Rd, Houston, TX 77054

[3]Present Address: Department of Chemistry, University of Pittsburgh, 3960 Forbes Ave, Pittsburgh, PA 15260

*Co-corresponding authors.

†These authors contributed equally.

1. Analytical spectra for synthesis of C_{60}-NOTA conjugate

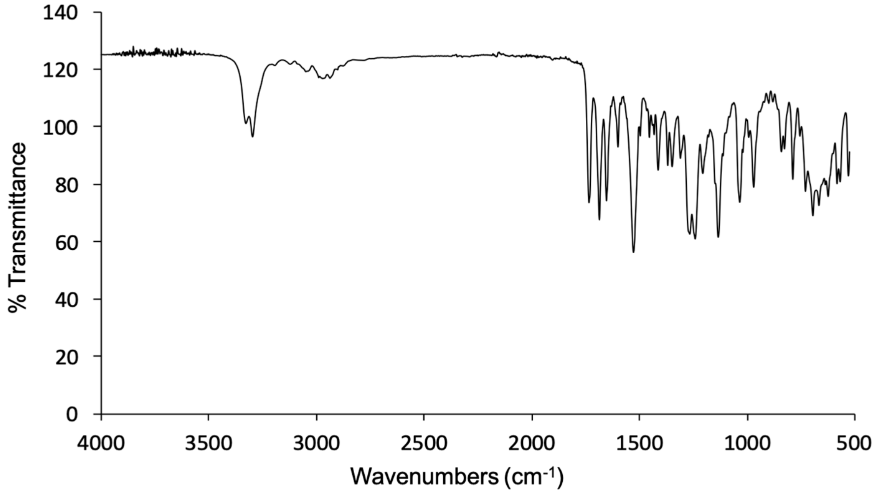

Figure S1. IR of compound 1.

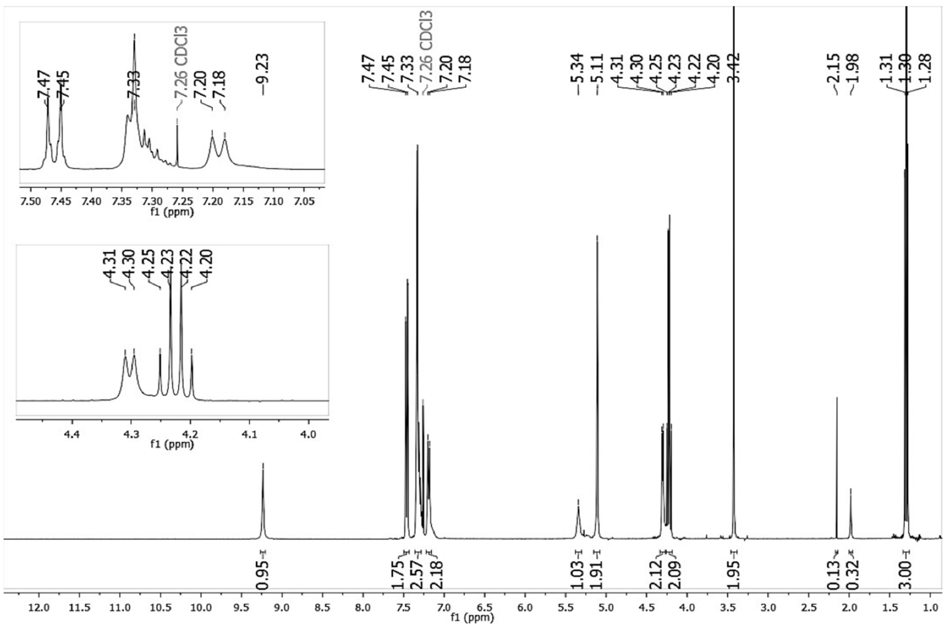

Figure S2. ^{1}H NMR of compound 1.

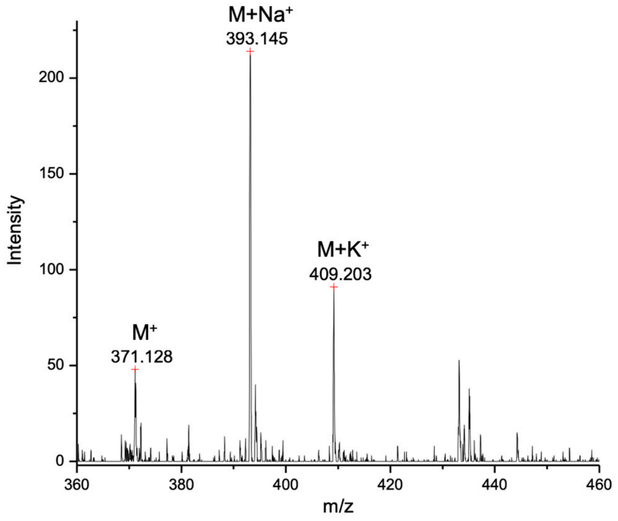

Figure S3. MALDI-MS of compound 1.

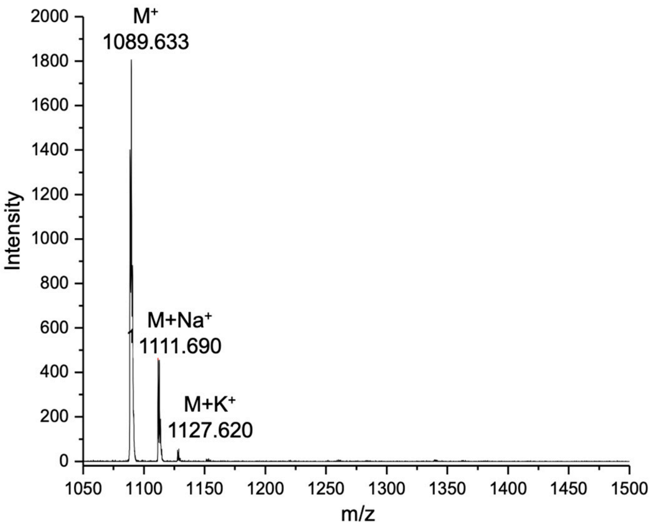

Figure S4. MALDI-MS of compound 2.

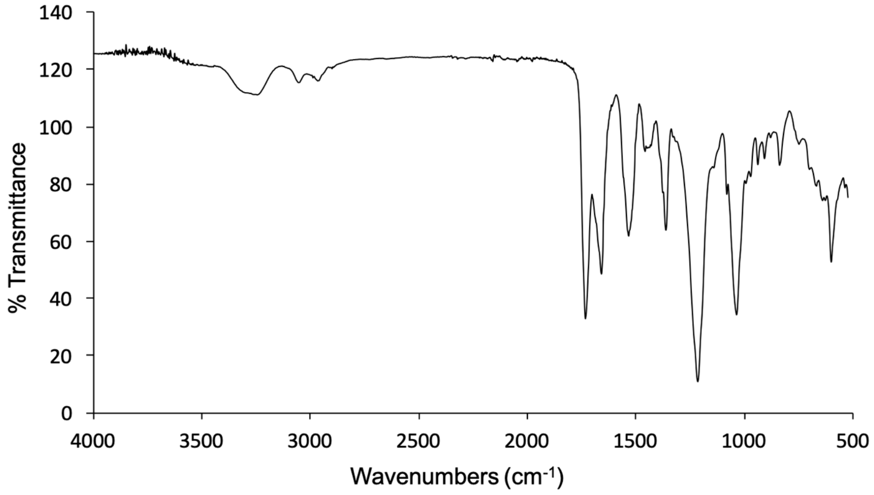

Figure S5. IR of compound 3.

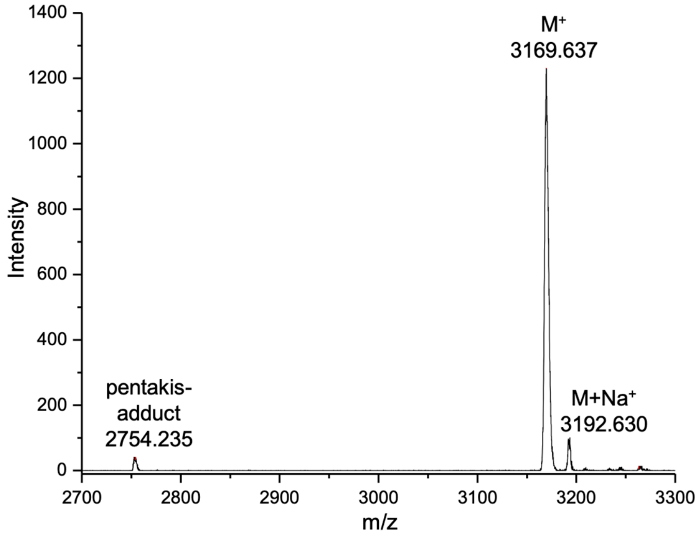

Figure S6. MALDI-MS of compound 3.

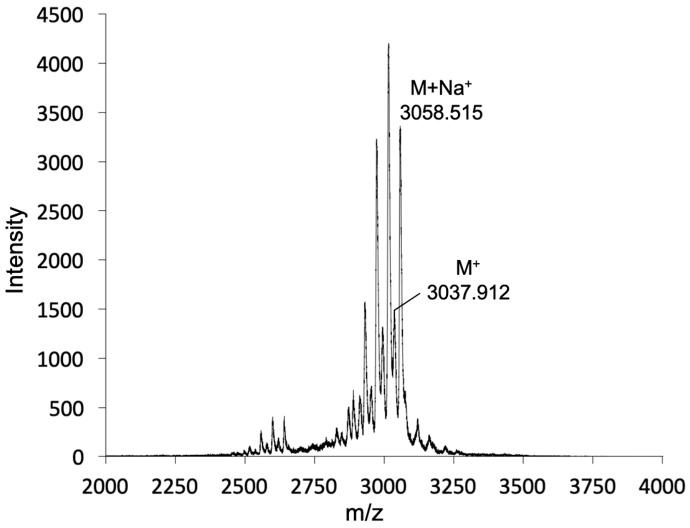

Figure S7. MALDI-MS of compound 4.

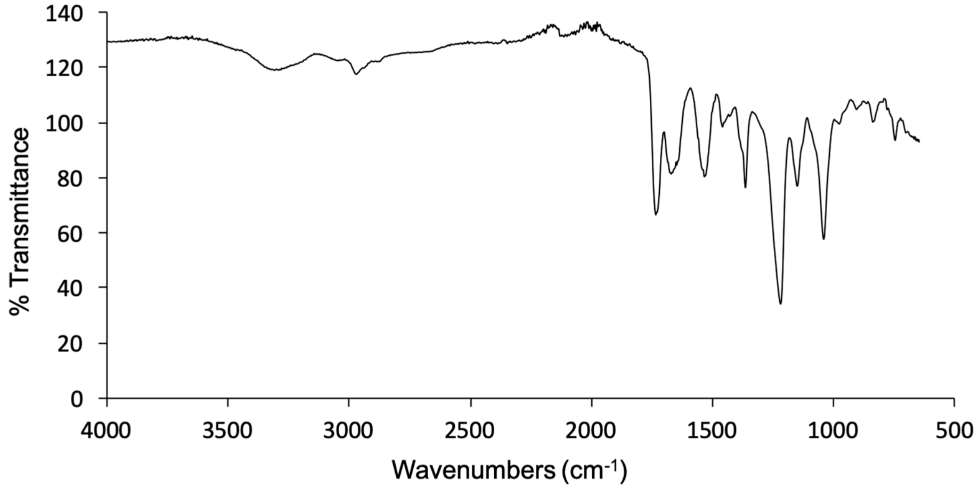

Figure S8. IR of compound 5.

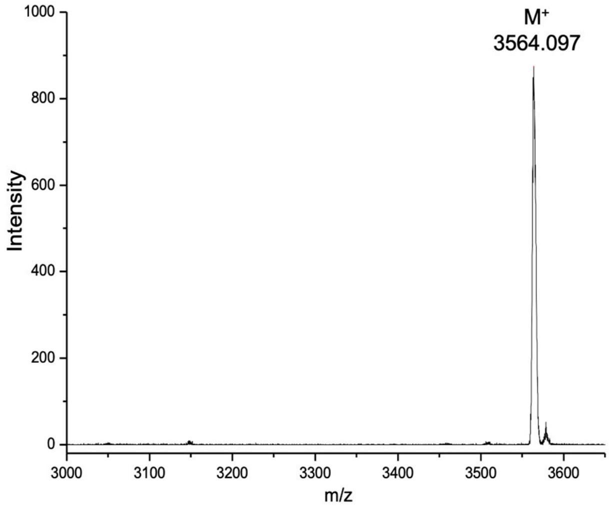

Figure S9. MALDI-MS of compound 5.

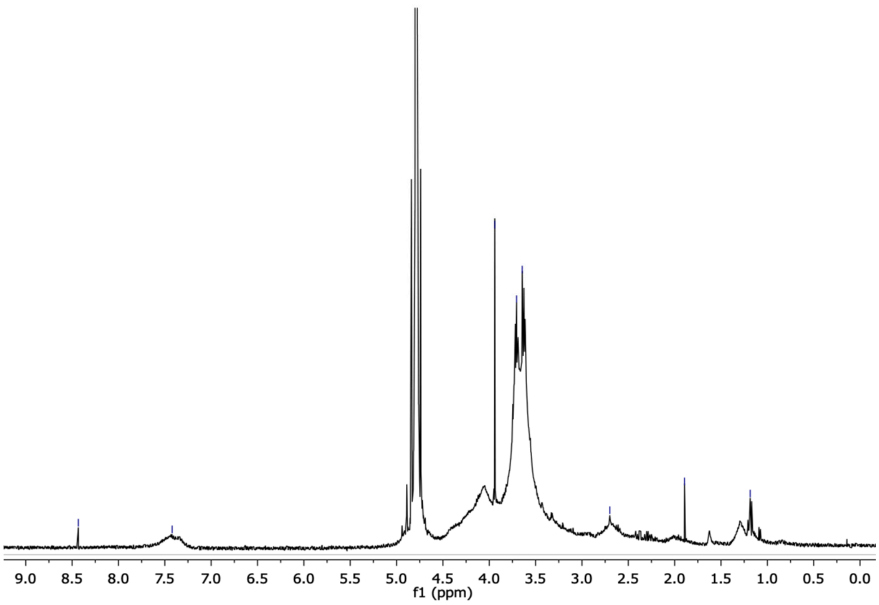

Figure S10. ^{1}H NMR of compound 6.

2. Characterization data for C_{60}-NOTA conjugate and C_{60}-[^{64}Cu]Cu(NOTA)

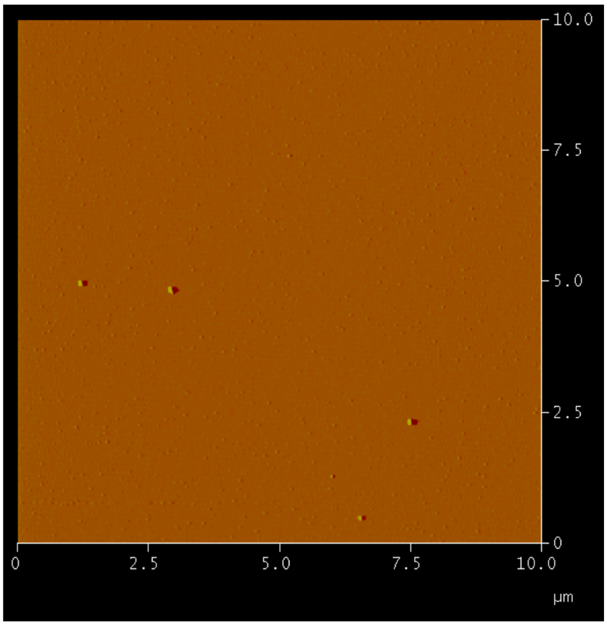

Figure S11. AFM of C_{60}-NOTA. Average horizontal distance of three pictured nanoparticles is 195 nm and average vertical distance of three pictured nanoparticles is 0.122 nm.

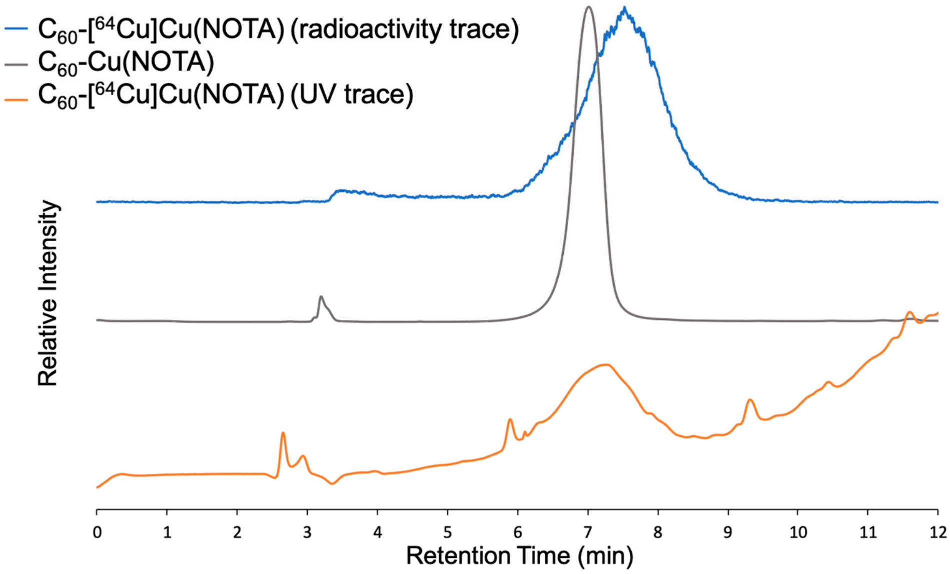

Figure S12. Characterization of C_{60}-[^{64}Cu]Cu(NOTA). Radio-HPLC trace of C_{60}-[^{64}Cu]Cu(NOTA) (blue, top), HPLC trace of C_{60}-Cu(NOTA) at 254 nm (gray, middle), and HPLC trace of C_{60}-[^{64}Cu]Cu(NOTA) at 254 nm (orange, bottom).

3. C_{60}-[^{64}Cu]Cu(NOTA) stability test data

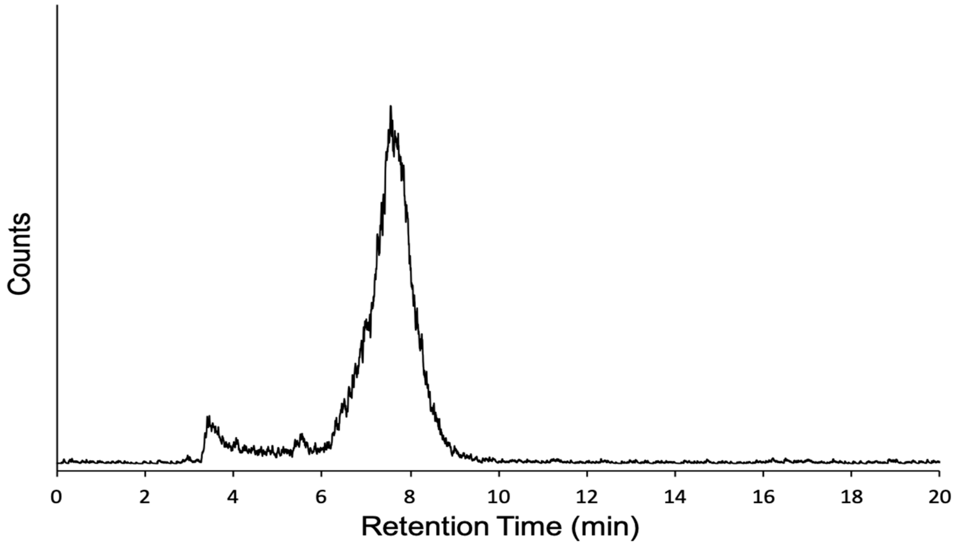

Figure S13. Shelf stability study. Radio-HPLC trace of C_{60}-[^{64}Cu]Cu(NOTA) after incubation in 1x PBS for 48 hours at room temperature.

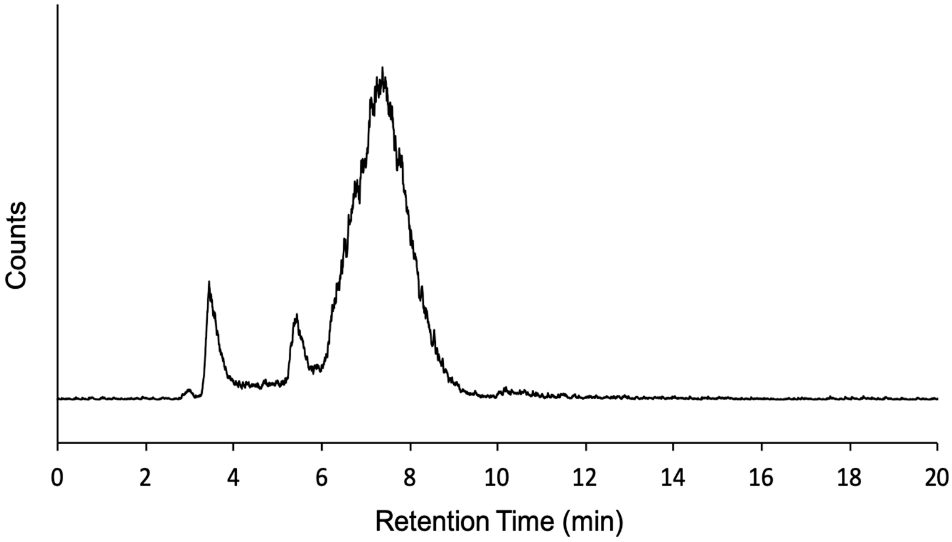

Figure S14. Shelf stability study. Radio-HPLC trace of C_{60}-[^{64}Cu]Cu(NOTA) after incubation in human serum for 48 hours at 37 °C.
